# Spectroscopic (UV/VIS, Raman) and Electrophoresis Study of Cytosine-Guanine Oligonucleotide DNA Influenced by Magnetic Field

**DOI:** 10.1371/journal.pone.0149488

**Published:** 2016-03-21

**Authors:** Seyedeh Maryam Banihashemian, Vengadesh Periasamy, Goh Boon Tong, Saadah Abdul Rahman

**Affiliations:** Low Dimensional Materials Research Centre, Department of Physics, University of Malaya, 50603 Kuala Lumpur, Malaysia; Aligarh Muslim University, INDIA

## Abstract

Studying the effect of a magnetic field on oligonucleotide DNA can provide a novel DNA manipulation technique for potential application in bioengineering and medicine. In this work, the optical and electrochemical response of a 100 bases oligonucleotides DNA, cytosine-guanine (CG_100_), is investigated via exposure to different magnetic fields (250, 500, 750, and 1000 mT). As a result of the optical response of CG_100_ to the magnetic field, the ultra-violet-visible spectrum indicated a slight variation in the band gap of CG_100_ of about 0.3 eV. Raman spectroscopy showed a significant deviation in hydrogen and phosphate bonds’ vibration after exposure to the magnetic field. Oligonucleotide DNA mobility was investigated in the external electric field using the gel electrophoresis technique, which revealed a small decrease in the migration of CG_100_ after exposure to the magnetic field.

## Introduction

In recent years, investigations related to the effects of various environmental conditions on DNA have been actively pursued through multidisciplinary studies due to the potential for versatile applications in biomedicine and electronics [1]. Although multiple research projects have covered a wide variety of responses, including electric field, magnetic field, electromagnetic waves, and acoustic wave effects on DNA, numerous potential applications in physics and medicine also exist in terms of the static magnetic field’s effect on DNA. The relationships between optical properties and delocalized charge in the DNA chain in different fields, such as a magnetic field, were also attractive due to the remote controlling and self-assembly use for bio-sensing and medical purposes [2,3,4,5]

DNA’s semiconducting properties with acceptable band gaps have been successfully used in optical applications, such as organic light emitting diodes (OLEDs) and organic field-effect transistors (OFETs) [6,7,8,9,10,11,12]. Primary researches on band gap analysis of oligonucleotides are new research in the last decades, carried out to answer specific questions about the electronic and optical properties and electron (hole) tunneling in DNA bases. It was speculated that DNA band gap is comparable to semiconductor band gap, useful in gene transition and cancer research. In 2001, Iguchi investigated the band gap of double strand of DNA using the tight-binding model[13,14]. Song et al. studied the effect of electrons on the band structure and the density of DNA states[15]. Electrical characterization of DNA fabricated in sandwich form (Al-DNA-Si-p) meanwhile was performed by Güllü et al. using a Schottky diode. Their results indicated that DNA molecules adopt semiconductor behaviour with a band gap of about 4.12 eV. The optical properties of self-assembled, super molecules of DNA have been characterized in thin-film form for use in electronics as organic semiconductors and for bioorganic purposes[16,17,18]. Svetlana Kilina et al. conducted theoretical band gap calculations using a different approximation method via density functional theory (DFT). Their results indicated that the band gaps of DNA bases had different values, ranging from 3.71 to 7.85 eV[19]. In a recent studies by our research group, electrical and optical characterization methods were utilized to determine the effects of magnetic field on DNA extracted from plants for chip application[20,21,22]. These published papers focused on studies on the behavior of electrical (current-voltage) and optical parameters (refractive indexes) of DNA under the influence of an external magnetic field [20,23,24,25]

In this research, diluted DNA oligonucleotides, cytosine-guanine (CG_100_), were subjected to an external magnetic field (250, 500, 750, 1000 mT) to study the magnetic field’s effect on the optical band gap and molecular vibration of CG_100_. Characterizations of CG_100_ were performed using ultra-violet-visible (UV-VIS) spectroscopy, Raman spectroscopy, and agarose electrophoresis gel. The results indicate that the applied magnetic field directly affected the phosphate and hydrogen bond, causing a redistribution of charge and increasing the optical band gap of the CG_100_ oligonucleotide. Though many properties of DNA under magnetic fields bear key interests to various research applications, this result can be used for the label-free detection of light or resistivity monitoring for medical engineering research purposes.

## Results

Aqueous suspensions of CG_100_ oligonucleotides DNA influenced by a homogeneous magnetic field (250, 500, 750, 1000 mT) were generated using two pairs of Helmholtz coils. UV-VIS and Raman spectroscopy and, gel electrophoresis were carried out for the magnetic field-exposed samples.

### UV-VIS Spectroscopy

**Fig 1:**
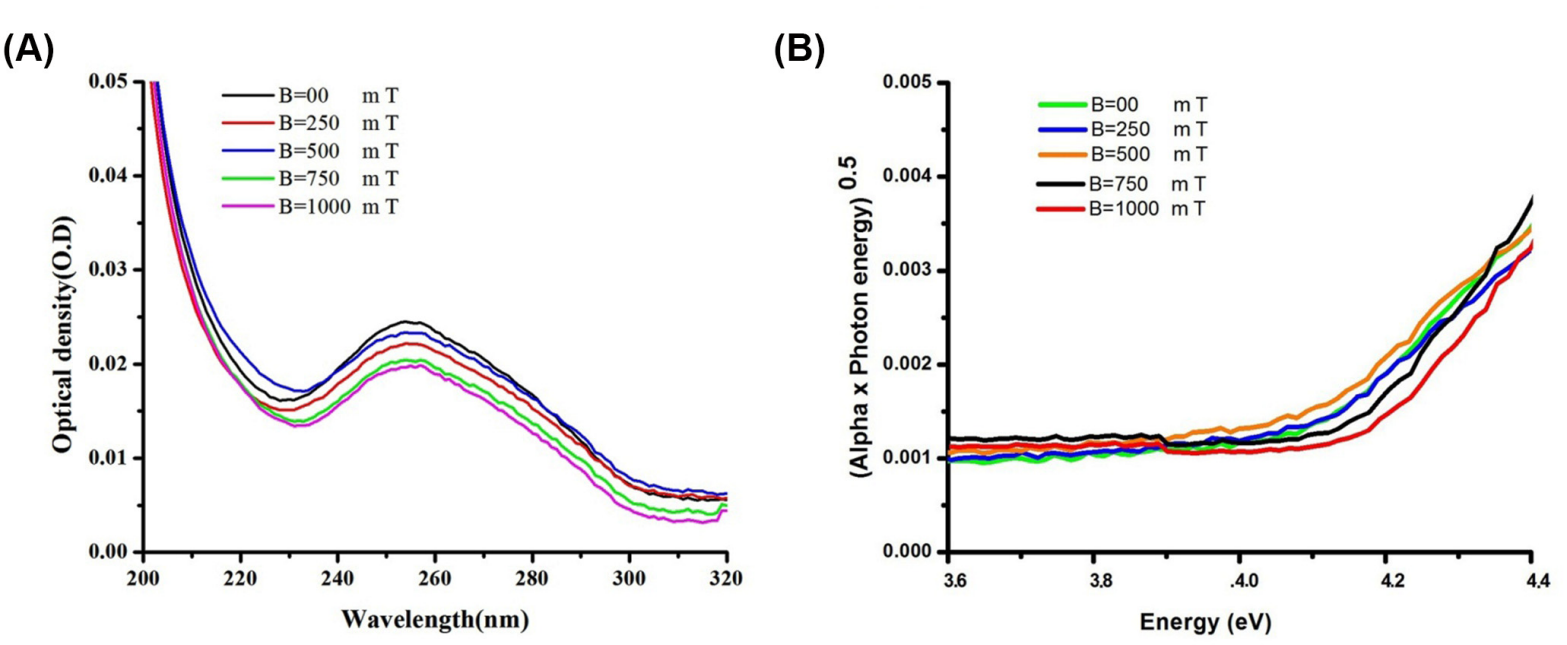
Absorption spectra and Band gap analysis of CG_100_. **(A)** The absorption spectra of diluted CG_100_ exposed to a magnetic field were fitted by the equations for direct band-gap transitions and (B) the inset shows (αE-photo) ^2^ versus photon energy for several magnetic fields for CG_100_ oligonucleotide DNA.

Fig 1 shows the UV-VIS absorption spectra of CG_100_ exposed to several strengths of magnetic fields (250, 500, 750, 1000 mT). The (αE-photo)^2^ versus E-photo evaluated for band gap analysis that α represents the absorption coefficient and E-photo indicate the photon energy [26,27](see S1 Text).

In this plot, extrapolation of the straight line to (αhν)^2^ = 0 axis gives the intercept, and therefore the value of the band gap can be extracted[28,29]. Table 1 shows the E_g_ values, which shows that the band gap increased, from 3.85 eV to 4.12 eV, by an improvement in the magnetic field strength by 1000 mT (see S2 Text).

**Table 1:** Comparison of the E_g_ values of CG_100_ determined versus several strengths of magnetic field.

Magnetic field	B = 0 mT	B = 250 mT	B = 500 mT	B = 750 mT	B = 1000 mT
**Energy Band gap (eV)**	3.85	3.86	3.75	4.02	4.12

### Gel electrophoresis

**Fig 2:**
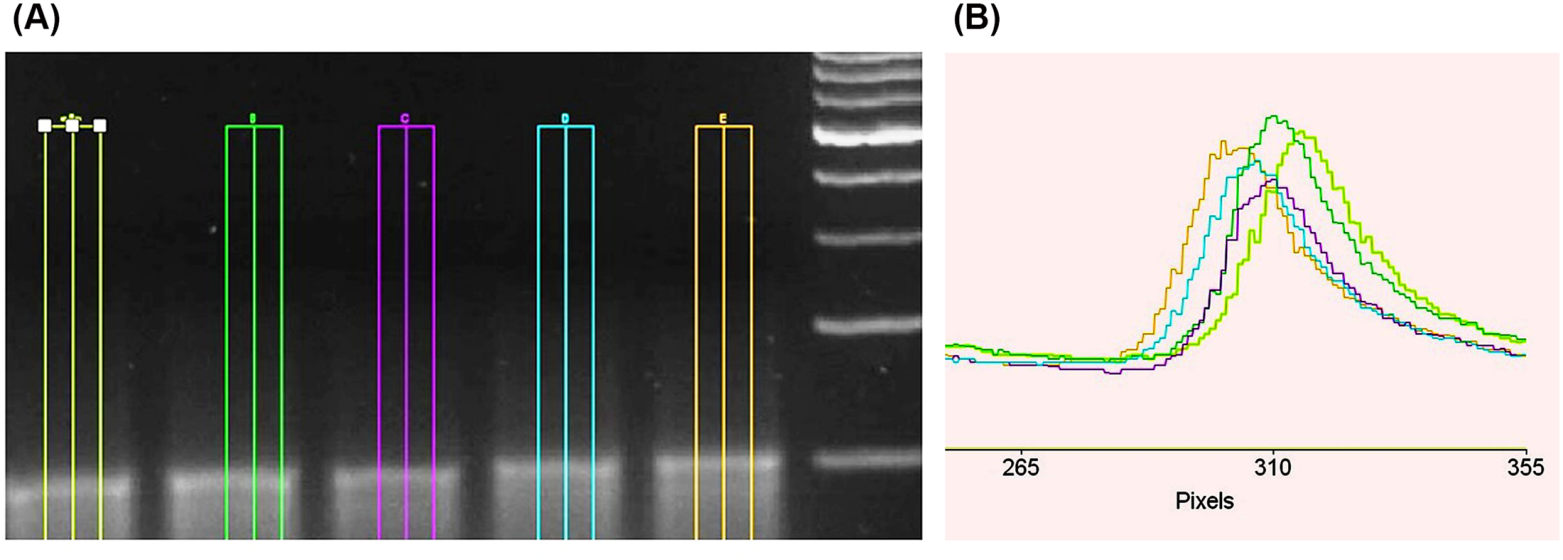
Gel electrophoresis image and analysis. (A) shows the gel electrophoresis image of five samples of CG_100_ after exposure to the magnetic field (0, 250, 500, 750, and 1000 mT) based on their displacement (d1, d2, d3, d4, and d5), respectively, and Fig 2B shows the analysis of CG_100_ displacement in the gel by evaluation of the intensity of the lane and lane position.

Fig 2a shows a gel electrophoresis image of CG_100_ influenced by the magnetic fields of 0, 250, 500 700 and 1000 mT. As depicted in the Fig 2a (gel pattern) and Fig 2b (data analysis of gel pattern), there is a small change in the DNA travelling in several magnetic field exposure. Displacement (shown by d1, d2, d3, d4, and d5 in Fig 2a and mobility of oligonucleotide DNA decreased by increasing the strength of the magnetic field. Fig 2b depicts the intensity of light emitted from DNA oligo versus the base distance on the pixel. The peak of the curve shifted to the left (as shown by the arrows) for CG_100_ in terms of low displacement and migration. As depicted in the Fig 2a and 2b, after 30 minutes of electrophoresis time, migration lengths are d_1_, d_2_ = 0.995 d1, d_3_ = 0.982 d1, d_4_ = 0.977 d1, d_5_ = 0.922 d1 correlated by the magnetic field 0, 250, 500, 750, and 1000 mT, respectively (see S1 Fig and S3 Text).

### Raman spectroscopy

**Fig 3:**
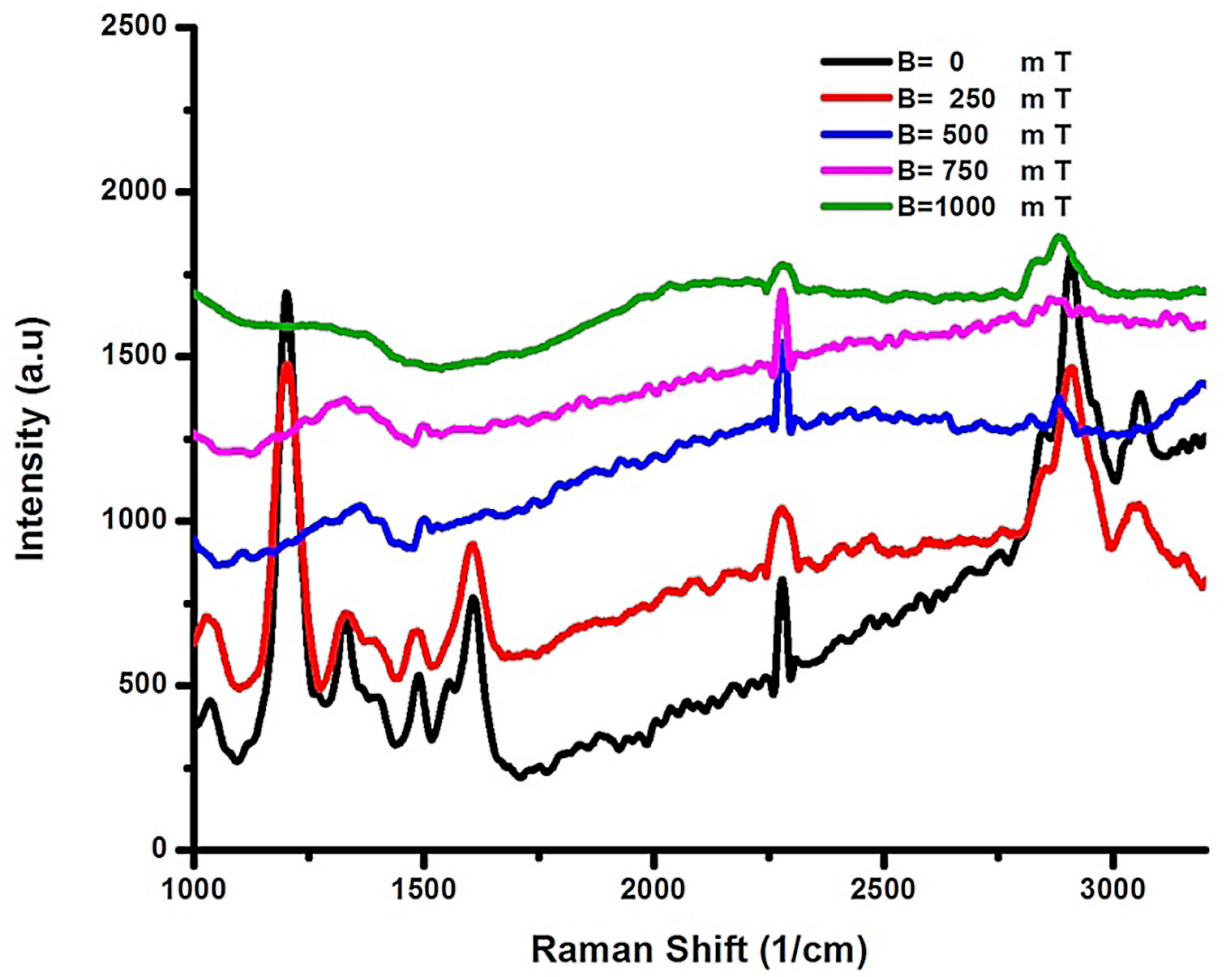
Raman spectra of CG_100._ Raman spectra of CG_100_ obtained using a 514 nm laser, before magnetic field exposure (B = 0) and after magnetic field (B = 250 mT, B = 500 mT, B = 700 mT and B = 1000 mT) exposure.

A 514 nm wavelength laser Raman spectroscopy was used to characterize the oligonucleotide DNA, CG_100_, in the presence and absence of a magnetic field. Fig 3 compares the Raman spectra of DNA before and after magnetic field exposure. The intensity change of the peaks in the 2700–3200 cm^-1^ and 1000–1700 cm^-1^ region was more prominent than in the rest of the regions. OH, NH and Phosphate group vibration peaks vanished after exposing to the magnetic field, 1000 mT (see S4 Text).

## Discussions

**Fig 4:**
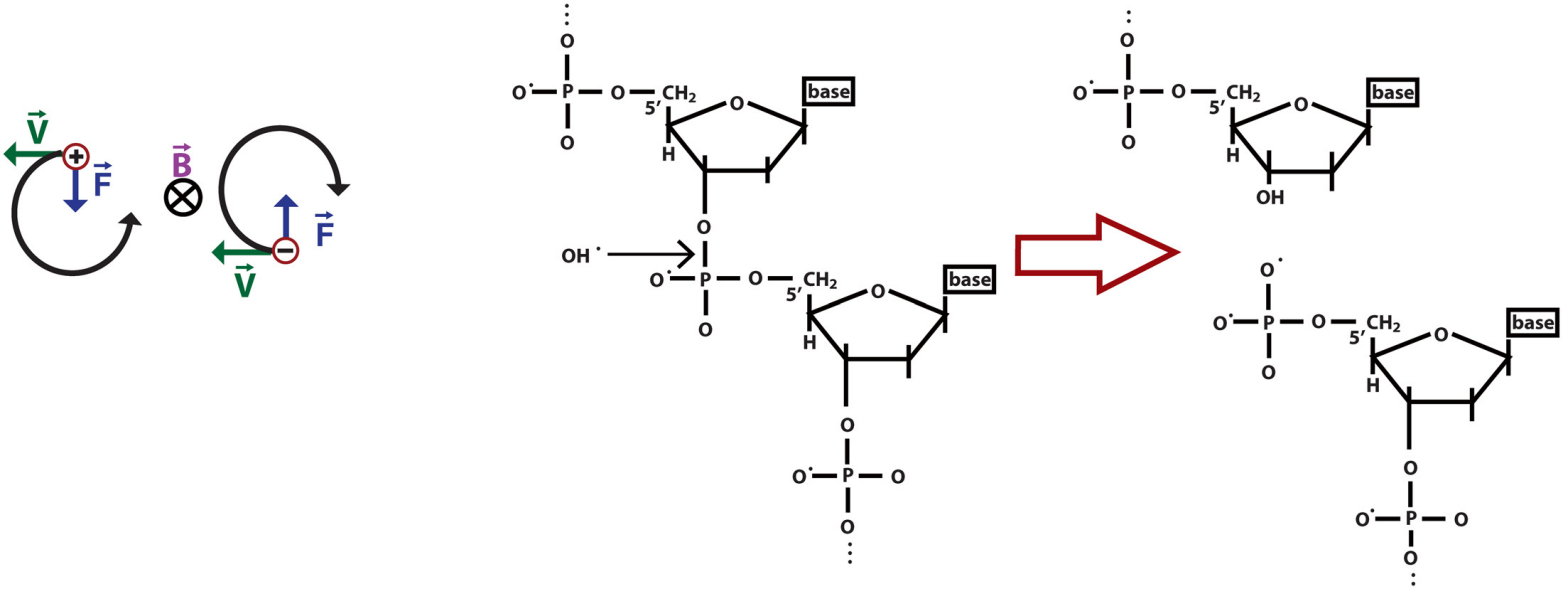
Lorentz force and DNA. (a) Diagram shows the Lorentz force F defined in terms of acting on a charge q that moves in a magnetic field with a velocity v. The F direction is the opposite for a positive charge versus a negative charge. Fig 4(b) meanwhile illustrates the interaction of DNA and the nucleophile hydroxide.

UV-VIS spectrum shows negligible peak shift in UV absorption under magnetic fields, which indicate little alteration of energy gap between the ground and first excited electronic states and also reflects significant change in absorption edge that make broader the peak shape by chemical reaction occurring to DNA under magnetic field. The E_g_ values, which shows that the band gap increased, from 3.85 eV to 4.12 eV, after influence by increase the magnetic field from B = 0 mT to B = 1000 mT (Table 1). Several unoccupied levels belong to phosphate group and water molecules located between the HOMO and LUMO bands of DNA. By transferring at least 0.2e of charge between backbone to bases regarding nucleophilic attach illustrated in Fig 4b and Lorentz force (Fig 4a), the energy level and band gap of DNA shift to the shorter wavelength [30].

The Raman shift of CG_100_ from 1000–3200 cm^-1^ is shown in the Fig 3; the collective H bond that vanished and shifted to the lower frequency (3000–3200 cm^-1^) is associated with OH and CH bonds. Deviation in the band position can also be the result of a break in the hydrogen bond and reorientation. Considerable variation in the intensity of the Raman band indicates a significant deviation with some chemical bond vibration in the 1200–1700 cm^-1^ region, including 1146 cm^−1^ phosphate backbone, C-N (1418 cm -1), 1240 cm ^-1^ (ν_as_PO_2_^-^), C-C (1290 cm-1) vibration region, and 1576 cm^−1^ stretching bonds in the guanine vibration region after exposure to several magnetic fields. The peaks at 1500–1300 cm-1 represent δ-CH and δ-NH vibration deviating from the initial band vibration of zero magnetic fields; normally, such decreases occur in cancer cells. This could be the result of the displacement of electrons, which affects the chemical bonds [31,32] and show that the interactions between biological samples and a static magnetic field occur via bound-ion dynamics. Exposure to magnetic field increases the polarity of the DNA molecules. The general mechanism is a type of nucleophilic attack on the phosphate side of the DNA helix and Lorentz force to turn charge transfer to the bases. Increasing the strength of the magnetic field change electrostatic polarities, band gap and vibration of bonds[2,33].

Gel electrophoresis method is described for quantitative analysis of DNA based on the difference between the rates of electrophoresis migration of samples in agarose gel. Electrophoresis gel is made of 2% of agarose gel adjusted to the sample size. As depicted in Fig 2a and 2b, after 30 minutes of electrophoresis time, the migration lengths are d1, d2 = 0.995 d1, d3 = 0.982 d1, d4 = 0.977 d1, and d5 = 0.922 d1 cm correlated by magnetic field (0, 250, 500, 750, and 1000 mT, respectively). By increasing the strength of the magnetic field, the movement of the oligomer decreased. Indeed, As a diamagnetic molecule, duplex DNA is not supposed to maintain magnetic field induced physical alteration after the external magnetic field is removed, then the major outcome from applying magnetic field on duplex is to induced backbone hydrolysis and to shift charges to nucleobases and chemical reaction occurring to DNA under magnetic field. Gel electrophoresis should show multiple bands but recombination of the exposed oligomers after a redistribution of charge shows slower mobility.

According to the band gap energy analysis extracted from the UV-VIS spectrum, Raman spectrum and gel electrophoresis results, one can conclude that magnetic field directly influences charge distribution and molecular bonding vibrations. Hydrogen atoms that make two close strands in the DNA strands and phosphate groups that manage the negative charge will be affected by the magnetic field.

## Methods

Liquid form of CG_100_ was characterized using spectroscopic measurements conducted in a controlled environment within a homogeneous magnetic field varied from 0–1000 mT. Micro chip electrodes were used to control the liquid temperature and the resistivity during the exposure to the magnetic field (explained in the chip fabrication section).

### Material

Two kinds of oligonucleotide DNA were used in this work. Cytosine -Guanine oligonucleotides DNA (CG_100_) were synthesized and purified by a small-scale PAGE purification. CG_100_, single strand oligonucleotide DNA with a molecular weight of 30859 g/mol, were used.

The purity of samples was measured using an amount of about 1.7–1.8, which is sufficient for the DNA analysis. Immediately after suspending samples in 1 mM EDTA and 10 mM Tris in a controlled pH of 7.5 to 8.0, pre-incubation of DNA oligonucleotide was carried-out. All samples were centrifuged for 2 minutes at 6000 rpm. The tube vortexes for 20 s. 5 to 25.0 μl of the DNA oligonucleotide were diluted and held under the magnetic field for 10 min. A p-type Si wafer (orientation <100>) possessing a resistivity of 20 Ω-cm (MEMC Electronic Materials) was used as the substrate, while gold (Au) wire was of purity 99.999%. (Kurt J. Lesker Company, USA). Other necessary chemicals (C_2_H_5_OH, deionized water and acetone) and MicroChem’s SU8 photoresist and developer were supplied by Sigma Aldrich. Agaros gel used to evaluate electrophoresis analysis. All chemicals were used without further purification.

### Experimental setup

**Fig 5:**
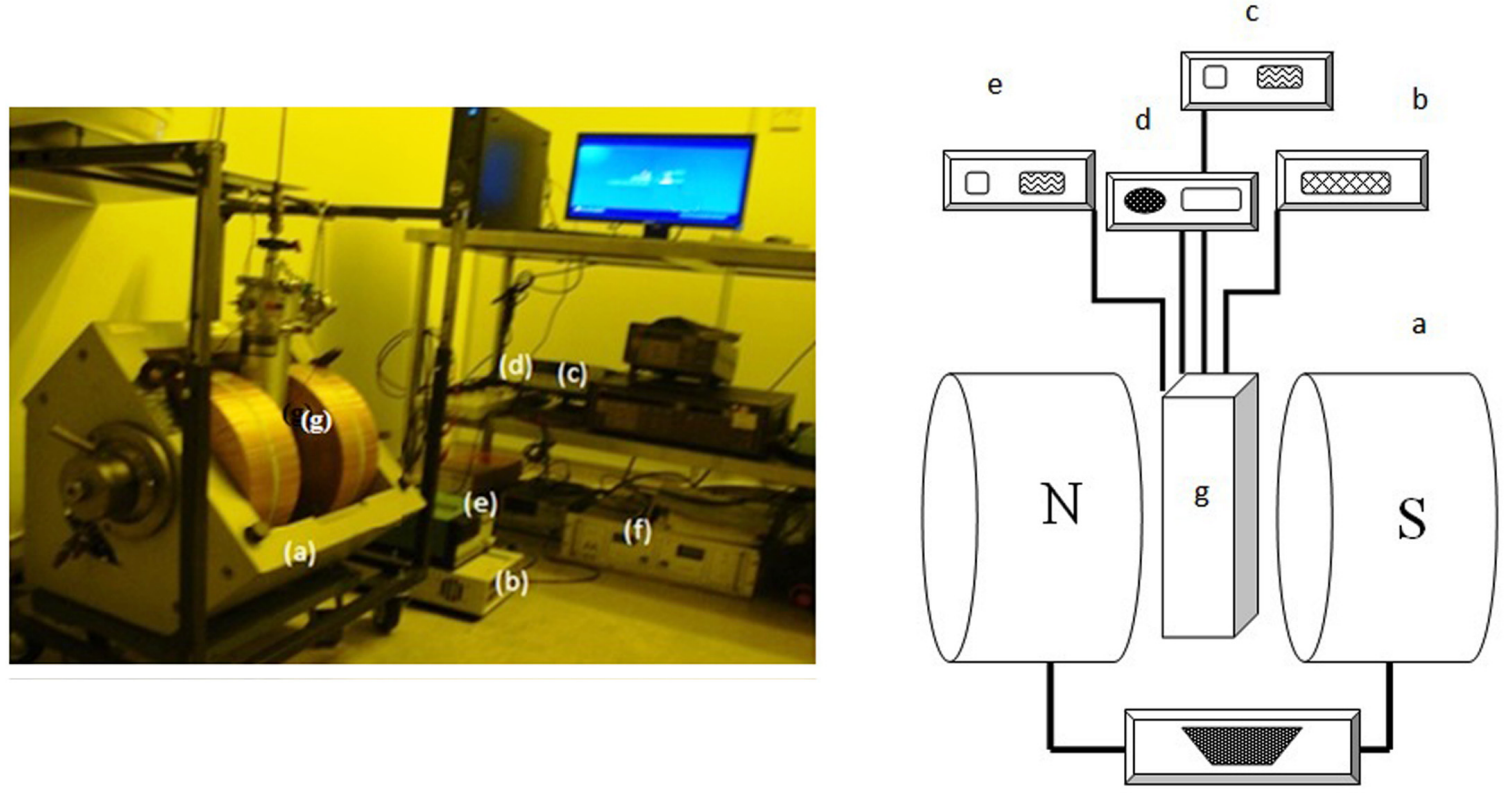
DNA is placed inside the magnetic field. Prepared oligonucleotide DNA sample is placed inside the magnetic field. Components include (a) electromagnet, (b) thermometer, (c) multimeter, (d) timer, (e) Gauss meter, (f) electromagnet power supply and (g) DNA sample. Sub Fig reflects a schematic aspect of prepared set up.

Fig 5 and sub Fig 5 shows a real photo and schematic diagram of the experimental setup respectively. The magnetic field was generated and maintained within a pairs of Helmholtz coils with variable distance. Variable homogenous magnetic field was provided by feeding current through the coils using a 2500-watt DC power supply (4500D, Electromagnet 3472–50). The cell contains the oligonucleotide placed in the center of the poles and was held for 10 minutes at 25°C. A thermometer (Thermocouple Wires Type K) to measure the temperature, electrometer (Keithley 617) to measure resistivity, timer (to record elapsed time) and a Gauss meter (to measure the flux density) were utilized to control the physical parameters before and after exposure to the magnetic fields (250, 500, 750 and 1000 mT).

**Fig 6:**
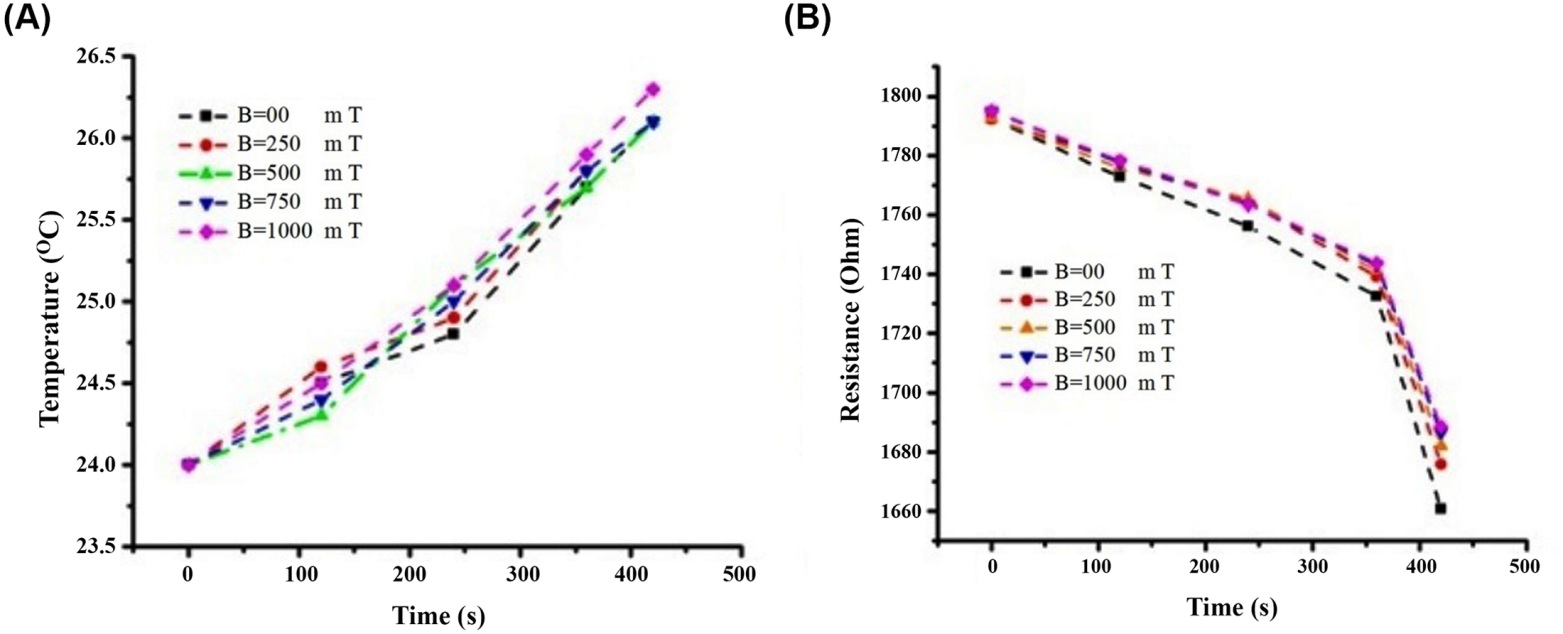
Magnetic field effect temperature and magnetic field of DNA. Localized temperature variation in various magnetic fields for CG_100_ oligonucleotide DNA (A) Graphs demonstrate the resistivity variation in various magnetic fields for CG_100_ oligonucleotide DNA (B).

UV-VIS and Raman spectroscopic characterizations were carried-out using PerkinElmer 750 and Ranishaw inVia micro-Raman spectrometer, respectively. Agarose gel electrophoresis was used to visualize the DNA oligonucleotide after influence by the magnetic field. The electrophoresis gel is made of 2% of agarose gel related to the sample size of about 100 mer. During exposure to the magnetic fields, temperature was measured by thermocouple wire. The resistivity was monitored by electrometer 617 during the magnetic field exposure using microchip electrode immersing in the diluted DNA. The temperature was changed by about 2–3°C after application of the magnetic field. Repeated measurements of all the samples were taken at a fixed temperature (25 ± 3)°C and resistivity (1795 ± 5) Ω to maintain a closed condition (as depicted in the Fig 6) (S5 and S6 Text).

### Fabrication of Chip

Two gold electrodes with gap size of about 50.0 μm were fabricated using UV-lithography process. SU8 photoresist was deposited onto Si wafer of dimensions (1.0 cm × 1.0 cm) after cleaning using standard RCA method. Spin coating was carried-out at a speed of 2000 rpm for 1 minute. In the next step, UV light was exposed through the designed mask via the lithography process. Post Exposure Bake (PEB) at 95°C was followed by the develop process using MicroChem’s SU-8 developer. Au deposition onto the sample was achieved using thermal evaporation with thickness of about 100 nm. The resulting thin film of Au was then annealed for 30 minutes at 200°C.

## Conclusions

The effects of magnetic fields on oligonucleotide DNA were investigated by spectroscopic and electrochemical methods; CG_100_ was tested based on spectroscopy experiments conducted in a controlled environment within a homogeneous magnetic field of varying strengths (250, 500, 750, and 1000 mT). The results demonstrated that the absorption of light in the UV (200–300 nm) region can be altered by changing the magnetic field. Observation showed that exposing DNA oligonucleotides to a magnetic field results in an increasing fluctuation of its optical band gap. Interactions between the DNA samples and a static magnetic field occurred via bound ion dynamics, as evaluated by Raman spectroscopy. The magnetic fields influenced the hydrogen (2700 cm ^-1^) and phosphate (1240 cm ^-1^ (ν_as_PO_2_^-^)) bond, as reflected in Raman spectroscopy, and increased the CG_100_ migration length in the gel electrophoresis. The capability of this method to remote control and even functionalize DNA oligonucleotides for bioengineering and medicine purposes is of interest for future application.

## Supporting Information

S1 Fig S1 Fig is supporting data for Gel electrophoresis image. (TIF)

S1 Text S1 Text is Supporting data for UV-VIS spectroscopy. (TXT)

S2 Text S2 Text is related to Band gap. (TXT)

S3 Text S3 Text is supporting data for Gel electrophoresis analysis. (TXT)

S4 Text S4 Text is supporting data for Raman spectroscopy. (TXT)

S5 Text S5 Text is supporting data for resistivity analysis. (TXT)

S6 Text S6 Text there is supporting data for temperature analysis. (TXT)
