# Supplementary figures and images for: Spectroscopic (UV/VIS, Raman) and Electrophoresis Study of Cytosine-Guanine Oligonucleotide DNA Influenced by Magnetic Field
Source: PLoS One. 2016 Mar 21;11(3):e0149488. doi: 10.1371/journal.pone.0149488 (PMC4801395; doi:10.1371/journal.pone.0149488)

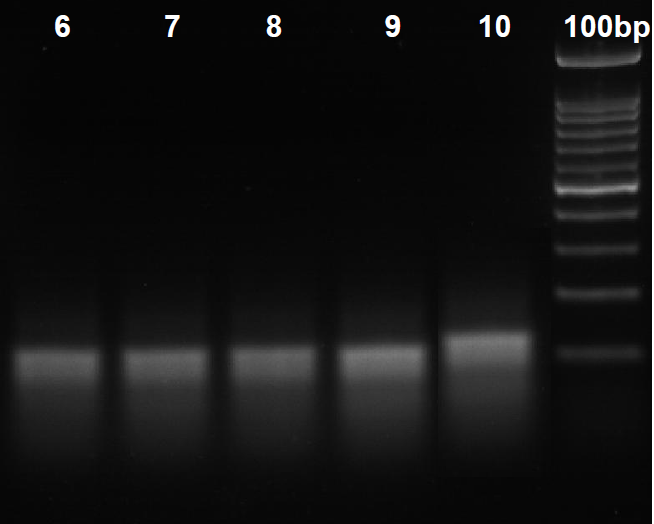

Supplement: S1 Fig — (TIF) [file pone.0149488.s001.TIF]
